# Attitudes and Perceptions Toward Healthcare Technology Adoption Among Older Adults in Singapore: A Qualitative Study

**DOI:** 10.3389/fpubh.2021.588590

**Published:** 2021-02-15

**Authors:** Sarah T. H. Low, P. Govind Sakhardande, Yi Feng Lai, Andrew D. S. Long, Satveer Kaur-Gill

**Affiliations:** ^1^Duke-NUS Graduate Medical School, Singapore, Singapore; ^2^Medical Social Services, Changi General Hospital, Singapore, Singapore; ^3^Office for Healthcare Transformation, Ministry of Health, Singapore, Singapore; ^4^School of Public Health, University of Illinois, Champaign, IL, United States; ^5^Department of Pharmacy, Faculty of Science, National University of Singapore, Singapore, Singapore; ^6^National University of Singapore, Singapore, Singapore

**Keywords:** digital disparities, health-information seeking, collectivistic technology use, social construction of health technologies, qualitative research

## Abstract

Smart Nation is a key initiative of Singapore to move toward digitalization of its industries including healthcare. The complex negotiations of aging amid Smart Nation are addressed in this paper, where we study the challenges faced to adapt the elderly for the digital revolution while ensuring dignified aging. While the healthcare industry accelerates its study and use of health technologies to improve diagnostics, treatment, and the quality of life of those in the aging category, the elderly socially construct these technological insertions that challenge the dominant understandings of what these technologies can do for their health outcomes. The study reveals re-constructions of these technological insertions through the voice of the elderly in their negotiations with health technologies in their everyday lives. Here, narratives reveal key themes that proliferate technology negotiation as barriers to everyday lived experiences.

## Introduction

The digital divide enhances health disparities for specific populations that face gaps in achieving technological literacy (1). The digital divide can be understood from the point of view of access as well as use. With metropolis cities like Singapore where the Smart Nation discourse centers the imaginaries of mobility and economic growth, certain segments of the population are left behind in negotiating technology use (2).

The Smart Nation initiative was launched by Singapore's Prime Minister in 2014. He visualized a Smart Nation to be one where “people live meaningful and fulfilled lives, enabled seamlessly by technology, offering exciting opportunities for all” (3). Digitalisation is seen as transformative, and essential to create a health ecosystem systematically embedded in technology (4–7).

Innovative technology to provide effective care to the elderly has been developed locally in recent years (8). Examples include assistive robotics by CHART, and Project SHINESeniors by the SMU-TCS iCity Lab, which enables elderly residents to age-in-place (9, 10). With a fast-rising aging population expected to reach 900,000 by 2030, it is pertinent to adapt to the elderly's growing needs, to ensure good quality of life and dignified aging (11).

### Digital Social Inequality

According to Tsatsou, creating opportunities for digital inclusion is critical especially with digital technologies potentially amplifying complex inequalities (12). These complexities include how different communities access technologies, use them, and benefit from them. Thus, with the acceleration toward digital societies, these gaps require greater interrogation. Studies reflect that for digital inclusion to be successful, a myriad of impediments must be addressed. This includes issues with costs and access to technologies (13). Furthermore, asset inequalities create further divides that limit technological adoption. Digital literacy relating to skills remains another challenge for various segments of the population to move toward digital inclusion (14). These structurally-centered barriers that exacerbate technological inequalities are studied using the frame of digital social inequality. Digital social inequality moves beyond the theoretical considerations of the digital divide. According to Halford et al. digital social inequality studies the divide beyond just access related issues, but also considers the “differential use of technology” as perpetuating inequalities (15). They elucidate this difference to specifically discuss various inhibitors of use, and argue that,

the critical distinction lies in possession of the appropriate resources to enable informed, effective, and secure use of ICTs including, for example, the skills to navigate the quality and quantity of information available effectively, to make enterprising use of information, to protect oneself from fraud and other potential harms and to use the knowledge and information accessed via the internet as a marker of social status (p. 939–940).

Factoring into account intersectional differentiation of use and how technology is socially constructed by different segments of the population (16). Therefore, there is a need to investigate the digital divide through an interpretive lens of differentiated use.

### The Elderly in Smart Nation

There is a significant need to alleviate issues of digital inequality and exclusion if we are to aim toward achieving a fully digital society, where each individual is expected to learn and use new technologies (5). Older adults face several barriers to technology adoption, more so if they are socioeconomically strained. For example, they report low technology usability, particularly if their age-related needs have not been sufficiently accommodated (17). This disproportionately impacts those with limited mobility, dexterity, and declining visual abilities. These users also reported data management and privacy concerns (17). Other barriers included high cost and low perceived ease of use of technology (18–20). Users were more likely to use technology if it enabled them to meet their needs. Despite initial receptiveness, certain users perceived activity monitoring technologies as inaccurate so they did not wish to continue using it (21). Technology acceptance by older adults is also affected by their perceptions of the uses of the technology and their proficiency in using it (19). Social networks also influenced attitudes toward technology, and could enable sustained technology use (19, 22).

With Singapore moving toward a Smart Nation and the surge of technological innovations for day-to-day use, some people are still left behind, resulting in a digital divide (23). Choi et al. found that in the United States, older individuals were less likely to use the Internet, and this was worsened by impairments to their activities of daily living (24). This particularly impacted elderly who were lower-income, of an ethnic minority and homebound; affordability of maintaining Internet subscriptions was also noted as a possible factor (24). This scene is reflected in Singapore. Lim et al. highlighted the presence of a socio-digital divide in multi-generational families with media-rich environments (1). The digital divide left behind vulnerable older adults who were ignorant of new technologies (1).

In this technological progression, it is critical that the aging population is not left behind. No studies have thus far explored factors influencing technology adoption among the aging population in Singapore. Therefore, our research question seeks to understand what the attitudes and technology adoption capacity of older adults are in order to bridge the digital divide?

In this project, we investigated the attitudes and perceptions of healthcare-related technology among older adults living in Singapore, in order to understand which technology insertions can translate to better outcomes for these individuals. The responses and findings were then used to develop personae to understand participants' receptiveness toward technology use and motivations to adopt technology. We then proposed practical recommendations for each persona, which could improve technology adoption rates and potentially bridge the digital divide.

## Methods

A qualitative exploratory research design was adopted to understand older adults' perceptions of healthcare-related technology. A qualitative approach was preferred over a quantitative one given the lack of research into the use of healthcare-related technology among older adults, especially in the Singapore context. Additionally, a qualitative approach would provide a more nuanced understanding, presenting the participants' perspectives, grounded in their specific social contexts (25).

In-depth semi-structured interviews were conducted with a total of 20 older adult participants. The inclusion criteria for participants were older adults aged 50 to 65 years living around Queenstown, in which Singapore's first public housing blocks were built in 1961, and hence has a sizeable elderly population. We specified the age range as such to get a sensing of the future elderly population, as part of the Alexandra Campus Masterplan 2030, as these individuals will then be aged 60 to 75 years. Seventeen participants were recruited through snowball sampling from senior centers. As theoretical saturation in the data had not been reached, convenience sampling was done to recruit three more participants in the Queenstown area.

Each interview lasted around 1 h. Interviews were based on an interview guide developed from our literature review (Appendix in Supplementary Material). Before the interview, the research objectives and rights of participation was explained to all participants. Permission to audio-record the interview was also sought. All participants (*n* = 20) gave written consent for their participation. Their identities have been protected using pseudonyms. Nineteen interviews were conducted in English, and one was conducted in Malay.

Thematic analysis was used to identify and analyse patterns within qualitative data (26, 27). Two levels of analysis were done: explicit and interpretive (28). Both levels are essential for a comprehensive analysis. The ten sub-themes were generated based on the six phases of thematic analysis according to Braun and Clarke, before being grouped into four broader, overarching themes (Table 1) (29).

**Table 1:** Phases of thematic analysis (29).

	**Phase**	**Description of the process**
1	Familiarising with own data	Transcribing data, reading and re-reading the data, noting down initial ideas.
2	Generating initial codes	Coding interesting features of the data in a systematic fashion across the entire data set, collating data relevant to each code.
3	Searching for themes	Collating codes into potential themes, gathering all data relevant to each potential theme.
4	Reviewing themes	Checking if the themes work in relation to the coded extracts (level 1) and the entire data set (level 2), generating a thematic “map” of the analysis.
5	Defining and naming themes	Ongoing analysis to refine the specifics of each theme, and the overall story the analysis tells, generating clear definitions and names for each theme.
6	Producing the report	The final opportunity for analysis. Selection of vivid, compelling extract examples, final analysis of selected extracts, relating back of the analysis to the research question and literature, producing a scholarly report of the analysis.

After transcribing the interviews, open coding was done. This required the researchers to summarize the participants' responses into codes. Similar patterns of behavior or accounts were stored in “nodes,” which are developed within and across transcripts. These were then categorized and recorded as themes and sub-themes. This process was cyclical and ongoing since new themes developed alongside new understandings gained from the data.

This study was approved by the Departmental Ethics Review Committee from the National University of Singapore, Chua Thian Poh Community Leadership Center (ref: CLC-DERC-19-0008). Written inform consent was taken for every study participant. All records are retained at Chua Thian Poh Community Leadership Center after the conclusion of the study.

## Results

### Participant Characteristics

Twenty older adults were interviewed. Their median age was 60.5 years (IQR: 56.5–62.0), with only 3 participants in their early 50's. Our participants were racially representative, with 13 Chinese (65.0%), 4 Malay (20.0%), 2 Indian (10.0%) and 1 Eurasian (5.0%). Less than half of our respondents were male (35%), and most participants either had a primary level or secondary level education. Seven of our participants were unemployed and among participants who worked, the median estimated income was $2,000 (IQR: $1,000-$5,000). Table 2 summarizes these demographic characteristics.

**Table 2:** Participants' demographic characteristics.

**No**.	**Age (years)**	**Gender**	**Race**	**Estimated monthly income (SGD)**
1	53	Female	Malay	0
2	58	Male	Chinese	1600
3	59	Female	Indian	400
4	65	Male	Malay	1,000
5	63	Male	Chinese	0
6	61	Male	Chinese	0
7	61	Female	Chinese	2,000
8	60	Female	Chinese	0
9	52	Female	Chinese	7,000
10	62	Male	Chinese	6,000
11	62	Male	Malay	2,000
12	65	Female	Eurasian	0
13	51	Female	Chinese	5,000
14	61	Male	Malay	0
15	60	Female	Indian	0
16	62	Female	Chinese	5,000
17	56	Female	Chinese	2,000
18	57	Female	Chinese	1,200
19	61	Female	Chinese	1,000
20	55	Female	Chinese	1,000

### Themes

Four key themes and 10 sub-themes were determined from the participants' responses.

#### Health-Seeking Behavior as Not Technology-Centered

Health-seeking behavior is defined as “any action or inaction undertaken by individuals who perceive themselves to have a health problem.for the purpose of finding an appropriate remedy” (30, 31). In this study, participants' health-seeking behavior entails seeking care from medical professionals, general health information seeking, and lifestyle choices to maintain one's health (32).

For our participants, availability of technology was not critical in determining their use of healthcare services. There were two main reasons for this - accessibility to health services was not dependent on technology, and there was no perceived need for health-related technology.

##### Accessibility of Healthcare and Health Information Is Technology-Independent

There were three reasons suggested why accessibility of health services was independent of technology. Firstly, face-to-face interaction with healthcare professionals was preferred. As Participant 4 noted, because of her age, it is “easier. to go to the hospital than using all this tech.” According to Participant 9, “I think [symptoms and progression of medical condition] can only be revealed through dialogue.” Secondly, medical professionals are seen as reliable sources of health information. Participants 1 and 4 both considered the doctor as the most reliable source, although they also seek general health information from the TV and newspapers. Thirdly, web searches were only used to supplement existing health knowledge or for general health information. Healthcare professionals were still the primary source for health information. For Participant 7, although most health information was obtained from online news websites, she felt that “if it is from the ministries, [she finds] it reliable.” Participant 9 also expressed that she usually refers to “SGH, the local websites, the hospital websites” for health information.

##### Lack of Perceived Need for Healthcare-Related Technology

Participants did not perceive a need for healthcare-related technology. These technologies might have been considered helpful, but they were not critical for daily life. According to Participant 3, she felt “like all [these health apps are] unnecessary” and said, “if I miss my appointment, I can just change another appointment at the doctor. Or hospital will message.” Participant 5 also shared that the “app [is] very troublesome.If [he is] sick, [he] will just go to the hospital.” Likewise, Participant 15 felt that it was easy to keep track of healthcare appointments because of SMS reminders, and did not need to use other apps.

#### Variable Influence of Social Network on Technology USE

##### Social Circle's Use of Technology as Possible Motivator for Own Technology Use and Vice Versa

Several participants mentioned that they know people who use health-tracking apps such as Healthy365 and SingTel StepUp. Participant 13, for example, relayed that her sister “recently started [to] use the SingTel one… for the free GB (gigabytes),” and she would occasionally go for walks with her sister and her friends. She also shared that her friends taught her how to use other functions of the Healthy365 app. Participant 16 shared that, being a Health Ambassador, she has been convincing her friends at church and her family to use the Healthy365 and HealthHub applications, and felt that her social circle regularly uses the applications.

##### Sharing of Health Information With Family and Friends

Most participants reported that they talked about their health within their social circles. Health was usually discussed with family members, except for those estranged from their family (Participants 3 and 15). Participant 8 expressed that she would regularly emphasize the importance of a healthy diet with her two sons. Participant 9 relayed that she would usually discuss her health information with her family, and Participant 11 discusses healthy lifestyle choices with his friends.

This informal sharing of health information among family and friends suggests a possible avenue for increasing social influence on health technology usage, and how it might reinforce familial ties and social roles.

#### Barriers to Health Technology Adoption

The participants also highlighted barriers faced in using mobile applications and wearable devices, and reservations regarding third-party health technologies.

##### Non-ubiquity of Smartphone Possession

Not all participants possessed smartphones, which suggests that there may be a population of older adults who will inevitably be unable to adopt health-related technology. For example, Participant 14 did not own a mobile phone. She keeps track of her polyclinic appointments using the appointment card. Sometimes her children remind her. However, she had no desire to obtain a mobile phone, citing cost as an issue, and not finding a need for it, as she could still contact her family using her house phone. Participant 12, who is disabled and unemployed, did not own a smartphone, citing “I can't get something that's smarter than me” and “I can't see small prints” as reasons. She could not use her current mobile phone due to technical issues and did not want to send it for servicing due to its cost.

Even among those who had a smartphone, most disclosed that mobile applications were not easy to use. For example, Participant 6 expressed difficulty in typing on a smartphone interface, which led to his disinterest in using mobile applications, saying that he takes “5–10 [min] to see one message on WhatsApp … Need to find the letter.cannot see the keyboard.” Similarly, Participant 7 also expressed frustration with the smartphone interface, sharing that it was “sometimes very irritating. connection no good, ask a lot of questions, issues when logging in. keep asking to key in [login details] …”, which made her find apps difficult to use.

##### Affordability of Applications, Devices, and Third-Party Healthcare Services

Participants who were open to using third-party healthcare services, such as home-based rehabilitation services, highlighted their concerns with service regulation and affordability. For instance, Participant 9, when asked about her thoughts on wearables such as Apple Watch and Fitbit, said, “Yeah I heard about it but it's expensive, and I'm not really that serious about counting my steps, so no I didn't think. I thought about buying Fitbit, but before I bought it, I knew of this free Step Tracker given to people who are 50 and above, I went to take it and therefore I won't buy it (FitBit).”

##### Privacy and Data Protection Concerns

When asked about her interest in sharing personal medical information with pharmacists, Participant 7 relayed, “No. regard health information as private.worried about leaking.” Participant 10 similarly felt that his “main concern…is how the information will be protected. Because…don't know will they go and misuse the information.”

#### Perceived Usefulness of Technology in Maintaining Health

Despite concerns, most respondents were receptive toward learning to adopt technology into their daily lives. This was discussed in relation to maintaining health goals, saving time, and sharing personal medical information with doctors.

##### Openness to Adopting Technology for Maintaining Health Goals

Participants highlighted that they were open to using technology to seek health information, citing Google searches and news articles as such channels. Participant 9 shared that, “when I research on the Internet it's more for the details of the particular [health problem] … So if I want more information I will go and search the web.” Likewise, Participant 10 said that he “[tends] to follow some health information in the news, media.”

Participants who used Healthy365 or similar applications (such as participants 8, 9, 10, 13, and 16) felt that the application was satisfactory in its purpose of tracking their number of steps. These individuals find these applications useful in maintaining their activity levels. Additionally, Participant 2 was open to using other kinds of technology in the future, “if it is simple to use.”

Technology was also perceived as useful for chronic disease management. Participant 13, who has high blood pressure, felt that the diet tracking option of Healthy365 was something she could use to track her cholesterol in the future. As for Participant 16, she uses a wrist blood pressure machine to self-monitor her blood pressure daily, to manage her hypertension.

##### Openness to Adopting Technology to Save Time

Participants 10, 13, and 16 highlighted their frustrations with polyclinic queue waiting times, and expressed a desire to see improved appointment-booking services that would save time, and that would be consistent across all polyclinics in Singapore. As Participant 10 relayed, “from the time you go [to make an appointment and see a doctor]. After you come out [it] is probably like 3 h. Very waste of time.”

Participants were generally receptive to the idea of teleconferencing. Participants 8 and 9 felt that it would be beneficial to patients who may not be able to travel to the hospital. Participant 4 indicated that this would be “convenient and fast.” However, some participants were concerned about the loss of personal touch.

##### Openness to Sharing Personal Medical Information With Doctors

Participants were also generally willing to disclose their personal medical information to their doctors. When Participant 6 was asked if she may have concerns with “sharing healthcare information through the app,” she replied that she was “okay with sharing with healthcare professionals only.” Participant 8 similarly responded that “if the doctor wants to know, of course I don't mind”.

## Discussions

Overall, our participants had a positive attitude toward technology in healthcare, but they did not perceive an immediate need to adopt healthcare-related technology. They did, however, express an openness to adopt these technologies to maintain their health goals and to use health services more efficiently, as long as affordability, personal data protection, and ease-of-use of the technologies were ensured.

Our findings on health-seeking and health information seeking behavior were supported by other studies. For health-seeking behavior, our participants seemed to only trust healthcare professionals, stressing the need for inter-personality in health-seeking but not in health-information-seeking behavior. Our participants highlighted that they preferred face-to-face interaction with healthcare professionals due to the presence of personal touch. Similarly, this is supported by previous study, which found that the elderly in the SHINESeniors project valued the human interaction that accompanied the technology more than the technology itself. Thus, other than changing the provision of healthcare, healthcare-related technology also changes terms of interpersonal communication (4). Instead of appreciating the functional benefits of healthcare-related technology, the elderly may be more concerned about losing the emotional benefits of human interaction (4).

Additionally, our participants found healthcare professionals to be the most preferred source for health information. This is reinforced by findings from another study on health information-seeking, which highlighted how doctors remained the “most highly trusted information source” despite new communication channels (33). Information-oriented sources, such as doctors, family and friends and newspapers, were also more trusted than entertainment-oriented sources, such as TV and radio (33). This illustrates how participants place different levels of trust in health information sought from diverse sources such as doctors, TV and radio.

We found that health-seeking behavior is largely technology-independent, and this behavior can be explained by several theories. Firstly, one reason why face-to-face interaction with healthcare professionals is still preferred among older adults may be due to “Continuity Theory.” As individuals age, they maintain a consistent pattern of behaviors and adapt in ways consistent with past behaviors (34). With the introduction of technology, there is a discontinuity in their environments, and they may experience a loss of control over their surroundings (34). This may be exacerbated with the onset of medical conditions, disabilities, or memory impairment, which are relatively common among older adults.

Secondly, our participants also perceived healthcare-related technology as unnecessary. One possible reason may be due to the paternalistic stance underlying Singapore's policies, whereby citizens accept a trade-off of some things, such as freedom of choice, if the government can meet their needs, particularly in the four areas of healthcare, education, housing, and employment, and provide a decent quality of life. This might explain our participants' general satisfaction with the current healthcare system. With their needs currently being met by the healthcare system, they might feel that adopting new healthcare-related technology is unnecessary. Perhaps, moving forward, we need to think of how to get individuals to take more ownership of their health, instead of relying on the state.

Additionally, older adults' ethnic and religious beliefs could also influence their attitudes toward healthcare-related technology. An Indian female participant restated multiple times that there was no need for all this technology and that she just left her health decisions to God. Her response is aligned with “Disengagement Theory,” which states how society and individuals prepare for death by mutually satisfying withdrawal from involvement with each other (35). This results in older adults decreasing their activity levels, seeking a more passive role, and interacting less frequently with others (35). For this participant, her choice to disengage was influenced by her ethnicity and religion (Indian Hindu). In Hinduism, as one ages, one disengages from early pressures, including pleasure-seeking or economically productive activities, and “meditates” till death. Thus, the enduring paternalistic stance of healthcare policies and the influence of religion and ethnicity leads to a disconnect between the macro level, in which there is a shift toward adopting technology to improve older adult's quality of life, and the micro level, in which individuals tend to rely on others instead of empowering themselves when it comes to health-seeking behaviors. Moving forward, as Singapore increasingly adopts sophisticated healthcare-related technology, a key policy consideration is understanding the role of culture and developing culturally-centered health infrastructures rather than uncritically imitating infrastructures driven by Euro-centric concepts of individualism, patient autonomy and privacy (2).

We found that our participants faced similar barriers to technology adoption as reported in the literature, especially privacy concerns (4, 17–19). The mixed feelings of older adults toward healthcare technology, such as apathy, fear, and disdain, raise questions about the underlying assumptions of health-care related technology (4). On one hand, technology such as activity sensors requires older adults to participate actively and be compliant with expected activity levels. On the other hand, failure to participate can lead to further exclusion. These two standpoints need to be reconciled to ensure the optimal and equitable use of such technologies (4). Our findings on the under-utilization of sophisticated healthcare-related technology are similarly reported by Peek et al. (19) Our participants who were open to using healthcare-related technology use simpler types of technology, such as smartphone applications, instead of complex and novel types of technology, like wearable devices and artificial intelligence (AI). This can be explained as older adults find it stressful to use new and unfamiliar types of technology (19). Furthermore, older adults are more likely to use intuitive decision-making, which can be characterized as automatic, associative and experiential; this requires limited processing resources, as older adults can rely on their internal schemas regarding products (19). As such, this type of decision making seems congruent with buying familiar products and not adopting new healthcare-related technology. While Singapore is moving toward the idea of a smart nation, we need to take deliberate measures to actively remove barriers to accessing technology, and include excluded groups, like the elderly, to avoid creating new forms of social division and inequality (4).

Our findings differed from the literature in one aspect–though we had expected participants' use of healthcare-related technology to be influenced by family, friends, and neighbors, we found that the influence of social networks on older adults' technology use was variable (2). Only some participants mentioned discussing healthcare-related technology with their family and friends and either influencing or being influenced by them. A few participants had grown distant from their children. Possibly, although older adults are willing to use technology if they have constant support while learning to use it, this support figure may be absent from their immediate social network. Our findings also did not clearly show that patterns of health information sharing followed collectivist values, such as filial piety and familialism, in contrast to Dutta et al. (2) Perhaps this difference in findings could be attributed to how most participants were recruited from social service agencies and were unemployed. Some were also receiving social assistance. Thus, other factors such as class, may have impacted the influence of one's social network on their use of healthcare-related technology. This would need to be examined in further research.

We had expected most participants to be averse to technology use, given their age. However, our findings showed that most participants generally perceived technology as useful in maintaining health, specifically in some areas. Some of them mentioned how they viewed online sources to seek health information, both for their own health conditions and general health. This supports Dutta et al. findings, whereby the Internet was viewed as a tool of empowerment for those able to use it–allowing participants to search for topics not commonly addressed by physicians such as alternative medicine (2).

Participants were also open to sharing personal medical information with doctors through technology, and adopt technology to save time and maintain their health goals. Interestingly, many participants used wearable activity trackers, such as the Step Tracker, to record one's physical activity. This was supported by a study, which reported that older adults perceive wrist-based wearable activity trackers as acceptable and useful, but need support in setting up the device and interpreting the data (36). The trackers are also attractive due to their ability to provide real-time feedback and enable easy monitoring and documentation (37, 38). Additionally, providing financial incentives to accompany such trackers can promote habit formation in terms of encouraging exercise (39). However, further research is needed to determine how desirable changes in health status can be sustainably maintained, and how devices can be effectively customized to older adults' needs (37, 39).

Furthermore, most participants tended to have limited understanding and knowledge about healthcare-related technology, specifically medication delivery, teleconferencing, and wearable devices. However, once the researchers explained what each technology entailed and how older adults would benefit from their use, they were more willing to try these technologies. This highlights a need for strong communication infrastructures between healthcare professionals and patients, to correct misconceptions and to promote the benefits of healthcare-related technologies.

As a whole, this study provides findings in these areas in the context of healthcare in Singapore, which has not been done before, although previous studies had explored the digital divide in the Singaporean context (1, 2, 11). Thus, this study affirms that the barriers to healthcare technology adoption observed in Western countries are similar contributing factors to the digital divide in Singapore (4, 17–19). This suggests that this may be a similar issue in other Southeast Asian countries. Our results and comparisons with other studies also suggest that social support, education on the use of healthcare technology, and ensuring new healthcare technologies are tailored to meet the varying needs of older adults, are critical to bridging this digital divide, and ensuring equitable adoption and use of healthcare technology among older adults in Singapore.

### Personae

Four personae were designed based on our findings, with regards to older adults' receptiveness and motivations for using healthcare technology. The personae were developed based on the interviews conducted and filtering how participants expressed technology through shared cultural behaviors and attitudes. Participants' responses on expectations and challenges, attitudes and behaviors to technology were coded for alignment and difference (40). Personae were developed by capturing their expressions and understandings of access and use from the interviews.

#### Persona 1

Fit and uses healthcare-related technology, is interested in learning about new mobile applications or health-tracking wearable devices.

#### Persona 2

Open to using healthcare-related technology but will only be interested in new health-related technologies if it is affordable and relevant to their daily needs. Currently uses technology they are familiar with.

#### Persona 3

Open to using healthcare-related technology but are only willing to use if someone is able to teach them how, and affirm they are using it right. Currently uses technology they are familiar with.

#### Persona 4

Does not use any healthcare-related technology, and does not see its value. Is resistant to learning about new technologies and prefers to continue using what is familiar to them.

#### Practical Recommendations

Campaigns such as the Active Ageing Programmes promoting the use of the Healthy365 application would target Persona 1 well. Additionally, to empower Persona 1 to manage their own healthcare, an integrated hospital record-keeping system would allow them to view their case files and make an informed choice on their treatment options. However, since Persona 1 is most receptive and willing to use technology, more attention should be directed to Persona 2 to 4 to bridge the digital divide.

For Persona 2, the concern of cost may be alleviated by setting up free-of-charge self-service booths in common neighbourhood areas, for managing health appointments and arranging medication delivery. These booths could be placed in Community Centres, Senior Activity Centres, void decks, and the like. Staff in these locations could educate older adults on how to use them.

In addition, key “community leaders,” such as regulars at community areas, or residents who interact frequently with neighbours, could be trained in technology use, so that they can educate their peer circles. These “community leaders” could act as supporting figures for Persona 3-type individuals. This could be done through existing digital clinics, or by establishing more of such clinics in new areas. Hospital staff could also take on the role of such “community leaders”; they could be stationed near common areas for gerontological patients. This will help in resolving any misconceptions older users may have regarding technology.

Since both Personae 2 and 3 are using technologies familiar to them, a systems-level intervention may be helpful in making their experience with current healthcare technology satisfactory. An integrated appointment-making system that allows patients to efficiently make, change or cancel appointments across any polyclinic or hospital in Singapore can be developed. This would reduce waiting times for patients as well.

For Persona 4, their health needs can be met by ensuring they have sufficient support from healthcare and community care workers. Since these individuals typically rely on healthcare infrastructure familiar to them, ensuring they receive the support they need in a new technology-based healthcare system would prevent them from being left behind in the digitisation of the healthcare industry.

One possible solution to encourage the use of wearable activity trackers among older adults, regardless of persona, is by providing a simple paper-based instruction manual, detailing set-up, use, and troubleshooting of the device (36). This would provide knowledge to older adults in a familiar medium, which may increase their likelihood of using that technology (36). Secondly, the design of wearable activity trackers can be catered to meet the needs of older adults. For easier viewing, displays could use large, high-contrast text with large light-on-dark letters and numbers (36). A waterproof design could be used to prevent damage in case the device is forgotten or accidentally damaged (36). Ideally, older adults themselves should participate in designing to achieve high acceptance rates (38). Thirdly, healthcare provision can be improved by documenting physical activity for healthcare professionals with these trackers, as older adults tend to highly value these professionals' advice (38). Lastly, these trackers could be made available in accessible locations like pharmacies, where older adults can learn more about them from pharmacists (41).

Although these personae characterize our participants, we should be mindful of not generalizing them to represent all older adults in Singapore. Older adults have diverse life experiences, values and knowledge and are situated in different contexts; all these factors can influence their attitude and current adoption of healthcare-related technology.

## Limitations

Firstly, our findings only explored the views of English-speaking older adults, as our inclusion criteria was limited to English and Mandarin-speaking older adults. Secondly, there might be a possible loss of meaning in translation, specifically for the interview conducted in Malay. To simplify complicated ideas such as “wearable devices” and “teleconferencing,” there may have been a high possibility of double translation or incorrect translation. Researchers may have unintentionally altered the meaning to make it more palatable for the participants to understand. This may have reduced the validity and reliability of the questions and in the process compromised the data collected. Thirdly, there was a lack of standardization among researchers as the interviews were semi-structured. Different types of follow-up questions were asked based on the elderly's responses and different examples were brought up to explain the same concept (i.e., AI). This elicited different responses from the participants.

Our future work in this area would seek to investigate the differences in demographics, attitudes, and health-seeking behaviours between older adults who do not use healthcare technology, and those who use healthcare technology regularly. As this study was a pilot study on the attitudes of healthcare technology adoption among older adults in Queenstown, we hope to expand our sample size in subsequent studies, to incorporate data from older adults in other heartland areas of Singapore, and across different socio-economic statuses.

## Conclusions

Our study findings show that technology is not a main driver of health-seeking behaviour among older adults in Singapore. Older adults in Singapore still prefer face-to-face interaction with healthcare professionals and may perceive technology as unnecessary, or do not have the means or know how to use it. Additionally, our findings stress the need to develop targeted solutions for elderly with different attitudes and usage levels of technology. The four personae give an insight into these targeted solutions. We can also tap on older adults' willingness to use technology in specific areas, such as seeking health information online, sharing personal medical information with doctors and maintaining health goals.

Older adults need to experience healthcare-related technology as easy to use and useful before they can accept it in their daily lives (42). To make healthcare-related applications and devices more enticing, we can also focus on their entertainment and aesthetic value (42). We also need to examine the influence of other factors, such as sociodemographic factors, on the use of healthcare-related technology. Sociodemographic factors, such as age, gender, and education, can influence one's experience of technology, ease of use and felt needs (43).

Within the smart nation discourse in Singapore, a challenge policymakers face is translating the “attractive but elusive imaginaries of smart city discourse” into “tangible interventions” (2). Until this challenge is addressed, the potential of healthcare-related technology in improving lived experiences of older adults will remain uncertain. To address ethical and practical challenges, a dialogue can be conducted among healthcare providers of long-term care homes and independent retirement communities, who work first-hand with older adults, and policy makers (44).

## Data Availability Statement

The original contributions presented in the study are included in the article/Supplementary Material, further inquiries can be directed to the corresponding author/s.

## Ethics Statement

The studies involving human participants were reviewed and approved by Departmental Ethics Review Committee, Chua Thian Poh Community Leadership Center, National University of Singapore. The patients/participants provided their written informed consent to participate in this study. Written informed consent was obtained from the individual(s) for the publication of any potentially identifiable images or data included in this article.

## Author Contributions

The authors have met criteria for authorship as stipulated by the International Committee of Medical Journal Editors (ICMJE), and have significant contribution to the study conceptualisation, design, execution, analysis, manuscript drafting and review. SL and PGS contributed to the design, data acquisition, analysis and interpretation of the findings, as well as drafting and revising the article. YFL, AL, and SK-G made substantial contribution to the conception and design of the study, as well as to interpreting findings, outlining, and critical revising of the article. All the authors have provided final approval of the submitted manuscript.

## Conflict of Interest

The authors declare that the research was conducted in the absence of any commercial or financial relationships that could be construed as a potential conflict of interest.

## References

[1] Sun Lim S Ling Tan Y. Old people and new media in wired societies: exploring the socio-digital divide in Singapore. Media Asia. (2003) 30:95–102. 10.1080/01296612.2003.11726709

[2] Dutta MJ Kaur-Gill S. Precarities of migrant work in Singapore: Migration, (Im)mobility, and neoliberal governmentality. Int J Commun. (2018) 12:4066–84. Available online at: https://scholarbank.nus.edu.sg/handle/10635/156272

[3] Au-Yong R. Vision of a Smart Nation is to Make Life Better: PM Lee. Straits Times (2014). Available online at: https://www.straitstimes.com/singapore/vision-of-a-smart-nation-is-to-make-life-better-pm-lee (accessed July 27, 2020).

[4] Kong L Woods O. The ideological alignment of smart urbanism in Singapore: critical reflections on a political paradox. Urban Stud. (2018) 55:679–701. 10.1177/0042098017746528

[5] Smart Nation and Digital Government Office. Smart Nation: The Way Forward. (2018). Available online at: https://www.smartnation.sg/docs/default-source/default-document-library/smart-nation-strategy_nov2018.pdf (accessed July 27, 2020).

[6] Basu M Rohaidi N. Exclusive: How Singapore Health uses AI to track its elderly care. GovInsider (2017). Available online at: https://govinsider.asia/innovation/bruce-liang-ihis-population-profiling/ (accessed July 27, 2020).

[7] Infocomm Media Development Authority (2018). Digital economy framework for action. Available online at: https://www2.imda.gov.sg/programme-listing/digital-economy-framework-for-action (accessed July 27, 2020).

[8] Wai C. The Future of Healthcare for Singapore's Ageing Population. Singapore Business Review (2018). Available online at: https://sbr.com.sg/healthcare/commentary/future-healthcare-singapores-ageing-population/ (accessed July 5, 2019).

[9] Smart Nation and Digital Government Office. Assistive Technology and Robotics in Healthcare. (2019). Available online at: https://www.smartnation.sg/what-is-smart-nation/initiatives/Health/assistive-technology-and-robotics-in-healthcare/ (accessed July 5, 2019).

[10] Choo F. New Tech for Old Folk to Live at Home Safely. Straits Times (2018). Available online at: https://www.straitstimes.com/singapore/health/new-tech-for-old-folk-to-live-at-home-safely (accessed July 27, 2020).

[11] Hoe SL. Defining a smart nation: the case of Singapore. J Inform Commun Ethics Soc. (2016) 14:323–33. 10.1108/JICES-02-2016-0005

[12] Tsatsou P. Digital inclusion of people with disabilities: a qualitative study of intra-disability diversity in the digital realm. Behav Inform Technol. (2019) 39:1–16. 10.1080/0144929X.2019.1636136

[13] Tsatsou P. Digital divides revisited: what is new about divides and their research? Media Cult. Soc. (2011) 33:317–31. 10.1177/0163443710393865

[14] Mubarak F. Towards a renewed understanding of the complex nerves of the digital divide. J Soc Inclusion. (2015) 6:71–103. 10.36251/josi.93

[15] Halford S Savage M. Reconceptualizing digital social inequality, information. Commun Soc. (2010) 13:937–55. 10.1080/1369118X.2010.499956

[16] Rashid AT. Digital inclusion and social inequality: gender differences in ICT access and use in five developing countries. Gender Technol Dev. (2016) 20:306–32. 10.1177/0971852416660651

[17] Wang S Bolling K Mao W Reichstadt J Jeste D Kim H-C Nebeker C. Technology to support aging in place: older adults' perspectives. Healthcare. (2019) 7:60. 10.3390/healthcare7020060 30974780 PMC6627975

[18] Elers P Hunter I Whiddett D Lockhart C Guesgen H Singh A. User requirements for technology to assist aging in place: qualitative study of older people and their informal support networks. JMIR mHealth uHealth. (2018) 6:e10741. 10.2196/10741 29875083 PMC6010833

[19] Peek STM Luijkx KG Rijnaard MD Nieboer ME van der Voort CS Aarts S. Older adults' reasons for using technology while aging in place. Gerontology. (2016) 62:226–37. 10.1159/000430949 26044243

[20] Hsiao C Tang K. Examining a model of mobile healthcare technology acceptance by the elderly in Taiwan. J Glob Inform Technol Manage. (2015) 18:292–311. 10.1080/1097198X.2015.1108099

[21] Fausset CB Mitzner TL Price CE Jones BD Fain BW Rogers WA. Older adults' use of and attitudes toward activity monitoring technologies. Proc Hum Fact Ergon Soc Ann Meet. (2013) 57:1683–7. 10.1177/1541931213571374 31263349 PMC6601605

[22] Neves BB Waycott J Malta S. Old and afraid of new communication technologies? Reconceptualising and contesting the “age-based digital divide.” *J Sociol*. (2018) 54:236–48. 10.1177/1440783318766119

[23] Toh E. The Big Read: Feeling Lost in a digital World, Some Elderly Shun Technology. TODAY (2017). Available onlineat: from https://www.todayonline.com/singapore/big-read-feeling-lost-digital-world-some-elderly-shun-technology (accessed July 5, 2019).

[24] Choi NG Dinitto DM. The digital divide among low-income homebound older adults: internet use patterns, ehealth literacy, and attitudes toward computer/internet use. J Med Internet Res. (2013) 15:e93. 10.2196/jmir.2645 23639979 PMC3650931

[25] Padgett DK. Does the glove really fit? Qualitative research and clinical social work practice. Social Work. (1998) 43:373–81. 10.1093/sw/43.4.373

[26] Lyons E Coyle A. Analysing Qualitative Data. London: Sage Publications (2008). 10.4135/9781446207536

[27] Rubin HJ. Rubin IS. Qualitative Interviewing: The Art of Hearing Data. Thousand Oaks, CA: SAGE (2004). 10.4135/9781452226651

[28] Willig C. Introducing Qualitative Research in Psychology, 2nd Edn. New York, NY: Open University Press (2008).

[29] Braun V Clarke V. Using thematic analysis in psychology. Qual Res Psychol. (2006) 3:77–101. 10.1191/1478088706qp063oa

[30] Olenja J. Editorial Health seeking behaviour in context. East Afr Med J. (2003) 80:61–2. 10.4314/eamj.v80i2.8689 16167716

[31] Oberoi S Chaudhary N Patnaik S Singh A. Understanding health seeking behavior. J Fam Med Prim Care. (2016) 5:463–4. 10.4103/2249-4863.192376 PMC5084583 27843863

[32] Mackian S Nafisa B Lovel H. Up the garden path and over the edge: where might health-seeking behaviour take us? Health Policy Plann. (2004) 19:137–46. 10.1093/heapol/czh017 15070862

[33] Lee ST Dutta MJ Lin J Luk P Kaur-Gill S. Trust ecologies and channel complementarity for information seeking in cancer prevention. J Health Commun. (2018) 23:254–63. 10.1080/10810730.2018.1433253 29436966

[34] Winstead V Yost EA Cotten SR Berkowsky RW Anderson WA. The impact of activity interventions on the well-being of older adults in continuing care communities. J Appl Gerontol. (2014) 33:888–911. 10.1177/0733464814537701 24942970 PMC5505561

[35] Cumming E Henry WE. Growing Old. New York, NY: Basic Books (1961).

[36] Mercer K Giangregorio L Schneider E Chilana P Li M Grindrod K. Acceptance of commercially available wearable activity trackers among adults aged over 50 and with chronic illness: a mixed-methods evaluation. JMIR mHealth uHealth. (2016) 4:e7. 10.2196/mhealth.4225 26818775 PMC4749845

[37] Maher C Ryan J Ambrosi C Edney S. Users' experiences of wearable activity trackers: a cross-sectional study. BMC Public Health. (2017) 17:880. 10.1186/s12889-017-4888-1 29141607 PMC5688726

[38] Seifert A Schlomann A Rietz C Schelling HR. The use of mobile devices for physical activity tracking in older adults' everyday life. Digital Health. (2017) 3:2055207617740088. 10.1177/2055207617740088 29942617 PMC6001246

[39] Shin DW Yun JM Shin JH Kwon H Min HY Joh HK. Enhancing physical activity and reducing obesity through smartcare and financial incentives: a pilot randomized trial. Obesity. (2017) 25:302–10. 10.1002/oby.21731 28063226

[40] LeRouge C Ma J Sneha S Tolle K. User profiles and personas in the design and development of consumer health technologies. Int J Med Inform. (2013) 82:e251–68. 10.1016/j.ijmedinf.2011.03.006 21481635

[41] Tedesco S Barton J O'Flynn B. A review of activity trackers for senior citizens: research perspectives, commercial landscape and the role of the insurance industry. Sensors. (2017) 17:1277. 10.3390/s17061277 28587188 PMC5492436

[42] Oksman V. Young people and seniors in Finnish “mobile information society.” *J Interact Media Educ*. (2006) 2006:2. 10.5334/2006-3

[43] Flandorfer P. Population ageing and socially assistive robots for elderly persons: the importance of sociodemographic factors for user acceptance. Int J Populat Res. (2012) 2012:1–13. 10.1155/2012/829835

[44] Demiris G Hensel BK Skubic M Rantz M. Senior residents' perceived need of and preferences for “smart home” sensor technologies. Int J Technol Assess Health Care. (2008) 24:120–4. 10.1017/S0266462307080154 18218177

