# Supplementary material for: Attitudes and Perceptions Toward Healthcare Technology Adoption Among Older Adults in Singapore: A Qualitative Study
Source: Front Public Health. 2021 Feb 15;9:588590. doi: 10.3389/fpubh.2021.588590 (PMC7917068; doi:10.3389/fpubh.2021.588590)
Supplement: Supplementary file 1 [file Data_Sheet_1.DOCX]

**Supplementary material**

**Interview Guide**

Name:

Age:

Gender:

Race:

Education Level:

Income Level: <500, <1000, <2000, <3000, <5000, <10000

(Collecting the information above will help us to analyse factors that may affect the use of technology among the elderly, common trends & cultural considerations)

A: Openness of elderly to sharing medical information & technology adoption

1. Would you be comfortable sharing briefly about your current health and well-being with us (the interviewers)?
   1. Follow-up questions may be: Do you have any chronic illness? Are you under any particular treatment / care plan? What have your experiences with the healthcare system been like? What were some services in the hospital/polyclinic/outpatient clinic that you feel were convenient, cost-effective, and helpful for you?
2. How do you currently manage your healthcare?
   1. How do you use electronic platforms (such as mobile apps) to access your medical information? Do you share your medical information with your loved ones? If so, how do you do so? Would you be comfortable using similar platforms to share your medical information with healthcare professionals outside of a hospital context?
      1. For example, would you be willing to share your medical history with a Unity / Watsons pharmacist for an accurate pharmaceutical prescription?
      2. Do you use HealthHub to record your physical activity (Tracker function), or MyHealth (under SingPass) to record or view your medical history (lab reports, health risk assessments, vitals data tracking, immunisation records)?
         1. If the respondent is familiar with MyHealth under HealthHub, can ask: Do you use your HealthHub account to share your medical information with your family and friends (and vice versa)? If so, how do you feel about this current service? What may be some things you like about this service, and some things that could be improved?
         2. If the respondent is not familiar: Would you be interested to learn how to use this, or similar services, to track your health? What are the current challenges you feel are preventing you from using this service more frequently? (For example, the respondent may comment about the usability / ease of use of the interface.)
         3. If the respondent is resistant to trying out new types of technology/services: What are your concerns, and what may be an ideal solution to these issues?
   2. Do you personally schedule and manage your medical appointments, or do your family members/social workers/volunteers help you to manage your appointments? Do you find it easy to manage your medical care? What kinds of services would you like to see that could make your healthcare management a more fuss-free process?
3. Current Information-Seeking Behaviour
   1. Who/Where do you seek health information from? (Eg. Newspapers/magazines, television, radio or Internet)
      1. Where do you get the most information from/feel that it is the most reliable?
      2. What kinds of health information do you seek?
4. How do you currently use mobile applications?
   1. What are the difficulties you face when you use mobile applications?
   2. What kinds of apps do you use, and why do you enjoy using them?
5. Do you use any mobile applications for healthcare purposes?
   1. Do you use the Health app included in your phone? (Or: do you use any health and fitness apps?) How do you use it/these apps? Do you consider them useful, and why?
   2. If the respondent refers to the use of Apple Watch or FitBits: How do you use FitBits or similar devices (do you regularly track your heart rate, physical activity level, sleep schedule, and the like)? Why do you enjoy using these services?
   3. Would you consider sharing your physical fitness and activity data with healthcare professionals, if this was a future function in these apps?
   4. What kind of mobile applications or services do you consider helpful?

B: Openness to Using Healthcare-Related Technology

1. Is using technology to manage your healthcare important to you?
   1. Do you think you may want to use technology to manage your healthcare in the future? Do you feel that using technology to manage your healthcare might increase your independence in your later years?
   2. Why is using technology to manage your healthcare important to you? (or: Why do you feel it is not important to you?)
2. How do you currently communicate with healthcare professionals?
   1. Would you be comfortable sharing your medical data (such as medical history, current and past medication, hospitalisation / referral records, immunisation records, and so on) with hospital care teams through an app? What may be some concerns that you have?
   2. What kind of technology applications would you like to see to improve your experience with the healthcare system?
3. Chronic disease monitoring
   1. Would you find it useful for you to monitor your health condition in the long-term using mobile health apps (that track your physical activity and vitals data)? Why or why not?
   2. If the respondent makes reference to use of wearable medical devices, or refers to a chronic condition that usually involves such devices: Are you currently using wearable medical devices, or any electronic devices in your home? How comfortable are you with using them, and why? What improvements do you feel can be made?
4. Teleconferencing & making healthcare payments/appointments online
   1. What are your feelings? Do you have any concerns?
      1. Explain that teleconferencing refers to communicating directly with a medical care team, receiving information on your medical reports and treatment
      2. If you have had teleconferences with a medical care team before, would you like to share your experience with us? Was it easy to use the teleconferencing platform? Did you have any privacy concerns? Do you feel that teleconferencing may be more convenient and/or cost-effective than physical trips to the hospital/clinic, and why or why not?
5. Use of AI to develop clinical triaging technology (eg: chatbots)
   1. How comfortable are you with using a chatbot to describe the reasons for your medical visit (such as your symptoms), and obtain a preliminary diagnosis? (This diagnosis may entail initial advice for self-management of minor issues first, before seeing a doctor physically.)

C: Influence of Social Network on Technology Use

1. What type of technology do your friends/family use?
   1. Do they use healthcare-related applications/technology?
   2. Do you talk about different application/technology with your friends/family?
2. Does their use of healthcare-related applications/technology affect your use?

D: Service procurement – how open are the elderly to buying healthcare services?

1. What is your experience in acquiring prescribed medication? Have you used any medication delivery or home-based rehabilitation services before?
   1. If not, do you think ordering of medication delivery services may be useful to you? If there were platforms for you to purchase care services (such as medication delivery, rehabilitation, or blood test services) online, would you be interested to buy such services? (Assuming that these services are made affordable to consumers.)
2. If you could choose to seek certain health services outside of the hospital, what types of services would you consider helpful for your needs?
   1. What may be your concerns? (Eg: Concerns about service quality, licensing & training of the healthcare professionals, cost, privacy concerns, especially if it’s a third-party platform such as Carousell)

**References:**

Elers, P., Hunter, I., Whiddett, D., Lockhart, C., Guesgen, H., & Singh, A. (2018). User requirements for technology to assist aging in place: qualitative study of older people and their informal support networks. *JMIR mHealth and uHealth*, *6*(6), e10741. doi:10.2196/10741

Peek, S.T.M., Luijkx, K.G., Rijnaard, M. D., Nieboer, M.E., van der Voort, C.S., Aarts, S., van Hoof, J., Vrijhoef, H.J.M., Wouters, E.J.M. (2016). Older adults' reasons for using technology while aging in place. *Gerontology*, *62*, 226-237. doi: 10.1159/000430949

Soja, E. (2017) Information and communication technology in active and healthy ageing: exploring risks from multi-generation perspective, *Information Systems Management*, *34*(4), 320-332, DOI: 10.1080/10580530.2017.1366217
